# Economic, social and demographic impacts of drought on treatment adherence among people living with HIV in rural South Africa: A qualitative analysis

**DOI:** 10.1016/j.crm.2022.100423

**Published:** 2022

**Authors:** Kingsley Orievulu, Sonja Ayeb-Karlsson, Nothando Ngwenya, Sthembile Ngema, Hayley McGregor, Oluwafemi Adeagbo, Mark J. Siedner, Willem Hanekom, Dominic Kniveton, Janet Seeley, Collins Iwuji

**Affiliations:** a Africa Health Research Institute, KwaZulu-Natal, South Africa; b Department of Global Health and Infection, Brighton and Sussex Medical School, University of Sussex, Brighton, UK; c Centre for Africa-China Studies, University of Johannesburg, Johannesburg, South Africa; d Division of Infection and Immunity, University College London, London, UK; e United Nations University Institute for Environment and Human Security, Bonn, Germany; f Institute of Development Studies, University of Sussex, UK; g Department of Health Promotion, Education & Behaviour, University of South Carolina, USA; h Harvard Medical School, Boston, MA 02114, USA; i School of Global Studies, University of Sussex, Brighton, UK; j Global Health and Development Department, London School of Hygiene and Tropical Medicine, London, UK; k Institute for Risk and Disaster Reduction, University College London, London, United Kingdom; l Department of Sociology, University of Johannesburg, Johannesburg, South Africa

**Keywords:** Climate change, Drought, HIV treatment adherence, Migration, Poverty, Social vulnerability, South Africa

## Abstract

The 2015 El Niño-triggered drought in Southern Africa caused widespread economic and livelihood disruption in South Africa, imposing multiple physical and health challenges for rural populations including people living with HIV (PLHIV). We examined the economic, social and demographic impacts of drought drawing on 27 in-depth interviews in two cohorts of PLHIV in Hlabisa, uMkhanyakude district, KwaZulu-Natal. Thematic analysis revealed how drought-enforced soil water depletion, dried-up rivers, and dams culminated in a continuum of events such as loss of livestock, reduced agricultural production, and insufficient access to water and food which was understood to indirectly have a negative impact on HIV treatment adherence. This was mediated through disruptions in incomes, livelihoods and food systems, increased risk to general health, forced mobility and exacerbation of contextual vulnerabilities linked to poverty and unemployment. The systems approach, drawn from interview themes, hypothesises the complex pathways of plausible networks of impacts from drought through varying socioeconomic factors, exacerbating longstanding contextual precarity, and ultimately challenging HIV care utilisation. Understanding the multidimensional relationships between climate change, especially drought, and poor HIV care outcomes through the prism of contextual vulnerabilities is vital for shaping policy interventions.

## 1 Introduction

Drought events challenge public health in many countries, contributing directly and indirectly to increasing mortality and morbidity through malnutrition, (non)infectious and chronic diseases, including mental illness (Stanke et al., 2013; Vins et al., 2015; Watts et al., 2017; WHO, 2019). Drought-related health impact mechanisms are often convoluted, operating through multidimensional systems to create negative outcomes for individuals and their societies (Berry et al., 2018; Orievulu et al., 2020; Vins et al., 2015). Droughts are recurrent in South Africa (Rouault and Richard, 2003) – a country battling with many infectious and noncommunicable diseases (NCDs) (UNAIDS, 2014). Drought exerts additional socioeconomic and health burdens, especially in poor rural areas characterised by longstanding structural inequalities, unemployment, HIV/AIDS and the increasing challenge of HIV drug resistance linked to sub-optimal treatment adherence among people living with HIV (PLHIV) (Derache, 2018).

In 2015, South Africa declared a national state of disaster due to the devastating effects of the El Niño-related drought being experienced across the country (Mare et al., 2018). Insufficient rainfall and extended dry spells caused widespread economic and livelihood disruption resulting in massive importation of food crops (like maize) to bridge resultant production shortfalls (Baudoin et al., 2017; Mare et al., 2018; Quinn et al., 2011). The ensuing disruptions impacted the health, wellbeing, and the quality of life of people, including PLHIV.

South Africa has the largest global burden of HIV and the largest ART programme with the Universal Test and Treat policy contributing immensely towards the reach of the ART programme (UNAIDS, 2014; Vandormael et al., 2019). The country is also deeply unequal as rising unemployment, especially among young people, exacerbates poverty, leaving only about 20% of citizens as middle class (Hanusch, 2018). uMkhanyakude district municipality exemplifies this condition.

Many South African studies on droughts, including the 2015 event, have examined the individual and country-level impacts on commercial as well as subsistence farmers (Mare et al., 2018; Mthembu and Zwane, 2017; Quinn et al., 2011). Some also critique drought planning, risk management policy implementation and relief systems, but with minimal outlook towards a drought-health (and illness) nexus (Baudoin et al., 2017; Mare et al., 2018; Mthembu and Zwane, 2017). The limited literature on health impacts has focused on farmers’ economic losses (Bahta et al., 2016; Hlahla and Hill, 2018; Quinn et al., 2011) and associated pain, anguish and possible mental (dis)stress – including stress of debt servicing culminating in cases of suicide and psychiatric hospitalisations (Mare et al., 2018).

Some studies have explored links between food insecurity and HIV prevalence in African countries. They suggest that food insecurity increases biological susceptibilities to HIV (Austin et al., 2021); that living with HIV can compromise one’s ability to grow or access food (Gillespie and Kadiyala, 2005a, Gillespie and Kadiyala, 2005b; Tsai et al., 2011); and, that food insecurity exacerbates a ‘sex for basic needs’ pathway whereby women are forced to engage in transactional sex and other forms of risky sexual behaviour in exchange for food or cash to buy food (Fielding-Miller et al., 2014; Gebremichael et al., 2018; Miller et al., 2011). Few studies have examined the relationship between drought and HIV-related outcomes specifically in the African context (Burke et al., 2015; Low et al., 2019). Burke et al., showed that across 19 African countries studied, an increase of up to 11% in HIV prevalence was positively associated with shortfalls in rainfall (Burke et al., 2015). Similarly, Low et al., demonstrated an association between drought and increased sexual risk behaviour such as early sexual debut, transactional sex and higher HIV prevalence – especially linked to external migration – in Lesotho (Low et al., 2019). These perspectives are further supported by a recent study (Austin et al., 2021) which draws upon data from 91 low- and middle-income countries to conclude that drought operates through food insecurity to heighten HIV transmission risk among vulnerable women in poor countries.

Burke et al. (2015) did not examine treatment adherence while Low et al. (2019) did not find an association between drought and reported ART use. Both studies were limited by their cross-sectional design, especially in the Lesotho study, which made it difficult to establish a causal relationship between their findings and drought. Against this backdrop, we examined the non-linear interactions between drought, socioeconomic and demographic factors and HIV treatment adherence among PLHIV in the Hlabisa sub-district, South Africa. Drawing on a systems approach (Berry et al., 2018; Peters, 2014; Vins et al., 2015), we analysed how drought’s multi-dimensional effects on interlinked socioeconomic and demographic factors potentially heightened challenges to optimal HIV care among PLHIV. We aim to demonstrate the multiple pathways by which the 2015 drought intensified existing challenges associated with HIV treatment adherence.

## 2 Conceptual Framework: The (complex) systems approach in health

Drought is one important feature of climate change and its diverse effects: floods, increasing temperature, rising seas, among others. Drought is categorised based on impacts such as the intensity of extended dryness due to insufficient rainfall resulting in ground water deficit (meteorological); reduced streamflow (hydrological); associated impacts on crop production linked to poor soil moisture and nutrient (agricultural); and resultant impacts on socioeconomic standing for people, communities and even governments (socioeconomic). The common denominator in these categories is constrained water supply (Rouault and Richard, 2003, 2005; Stanke et al., 2013). However droughts are described, there are associated consequences: reduced water quantity and quality, with impacts on soil water and nutrients including agricultural and livestock production (food insecurity); access to sufficient water for various human activities; and impacts on human health either due to insufficient access to portable clean water, poisoning of drought-resistant tubercrops like cassava (causing the Konzo disease) or through infections (Stanke et al., 2013). These ecological as well as health-related drought impacts contribute to a nexus of interrelated factors that were evident in uMkhanyakude, a context where PLHIV are also struggling with poverty. Although we recognise that seasonal water shortages are a chronic issue in uMkhanyakude, our study is specifically intending to interpret PLHIV’s experiences during and as a result of the 2015 El-Nino drought which was declared a drought emergency in 2014 (COGTA, 2018; Starkey, 2017) even earlier than the national declaration in 2015.

A feature of climate change and health dynamics including drought, is the complexity underlying the channels of impact and the flow of influence that constrain individual behaviour (Berry et al., 2018). To explain this complexity, systems thinking – the systems approach – has been employed in climate change-health nexus studies (Berry et al., 2018; Orievulu et al., 2020; Vins et al., 2015) to show and explore interconnectivities and complexities existent within and between variables in a multifaceted manner. It enables a clearer, though complex, representation of how interconnected variables are further linked and linkable to a distinctive set of outcomes (Peters, 2014). Specific to environmental stressors, like drought, some studies have used systems thinking to explore their nexus with health outcomes, through identifying intermediate factors that help to drive the different levels of influence or behavioural constraints that are arguably linked to different health outcomes (Berry et al., 2018; Orievulu et al., 2020; Vins et al., 2015).

We adopted this systems approach, embedded in the qualitative methodology, to explore, and possibly explain, pathways by which drought contributes to failing HIV care among PLHIV. The conceptual representation that we constructed drew on thematic analysis of interviews and existing ART adherence literature. The goal is to show the complexities underlying how the experience of the 2015 drought potentially contributed to an exacerbation of pre-existing contextual vulnerabilities and precarious livelihoods with potentially challenging effects on HIV care. Drought’s influence on interruption in HIV care cannot be explicitly demonstrated since there is no direct causal pathway between both variables. However, adopting this systems approach enabled us to represent and explore possible interconnections by recognising and identifying the intermediate factors triggered or exacerbated by drought. Although some of these factors represent the contextual vulnerabilities of PLHIV in Hlabisa, we argue that experiencing the 2015 drought was an additional stressor to already precarious livelihoods resulting in competing priorities, and trade-off of HIV care against pursuing economic sustenance.

## 3 Study setting

Hlabisa is a sub-district in uMkhanyakude, the second largest district municipality within the KwaZulu-Natal (KZN) province. uMkhanyakude has five local municipalities, uMhlabuyalingana, Jozini, Big 5 False Bay, Hlabisa and Mtubatuba, and is home to >600,000 people (Massyn et al., 2020). It is one of the most economically deprived districts in South Africa with pervasive poverty, unemployment and poor educational attainment (Massyn et al., 2020). Economic activities in this area centre on small scale agriculture, livestock breeding – especially cattle-keeping – public service, petty-trading and other forms of menial labour. Most households’ income are linked to public service, petty-trading, menial labour, government grants and pensions (Muhwava et al., 2010).

Zulu history shows that agriculture and livestock shape the socioeconomic and cultural context in this district – having been described as the central pillars of Zululand in the pre-colonial economic context (MacKinnon, 1999). The male-dominated cattlekeeping was noted to somewhat usurp agriculture partly due to the emergence of the White-settler colonialists, colonial legislation such as the 1936 Land Act and the Group Areas Act amongst others that not only expropriated lands from indigenous people, consigned them to reserves but also allocated only 13% of arable lands to indigenous populations – including the Bantu/Nguni peoples (Clark et al., 2007; MacKinnon, 1999; Shava and Masuku, 2019).

With the mining and industrial revolution as well as increasing white-owned private commercial farming, migrant/waged-labour became widespread (Collinson, 2010; Posel, 2004) and small-scale (subsistence) agriculture – which was itself under attack (MacKinnon, 1999) – became the remaining option for some people as land inequities (even in the reserves) exacerbated the plight of people. Consequently, cattle keeping became even more dominant in the immediate and post-colonial context of the Zulus mainly because of the centrality of cattle to their identity and overall way and philosophy of life (Shava and Masuku, 2019). This is exemplified in how Zulus perceive cattle as a measure of (generational) wealth (beyond money), hence the historical cattle-raids, and the centrality of cattle in settling (family) feuds, lobola (presenting cows as dowry to a bride’s family during marriage) and religious ceremonies amongst others (MacKinnon, 1999; Shava and Masuku, 2019). The shift to a monetised economy (of exchange) impacted negatively on many societies, including the Zulus (Shava and Masuku, 2019). The consequent widespread poverty among this people heightened rural-urban and circular migration (for waged labour) (Posel, 2004) – a phenomenon linked to high HIV prevalence in rural KZN (Camlin et al., 2010; Clark et al., 2007; Muhwava et al., 2010), currently estimated at 31.3% (NoamiModel, 2020). HIV/AIDS remains a leading cause of mortality especially among individuals aged 25–64 years (Massyn et al., 2020).

South Africa and indeed this northern KZN has recorded data on drought dating back to 1900 (Rouault and Richard, 2003, 2005), but the 2015 El-Nino-related drought was one of the worst in recent history (Baudoin et al., 2017; Corke and Whittles, 2015). This is because it combined components of all four kinds of drought – reduced average rainfall, depletion of groundwater reservoir and freshwater, destruction of agricultural production and lasted longer than 6 months to a year (Baudoin et al., 2017; Corke and Whittles, 2015; Stanke et al., 2013).

**Fig. 1:**
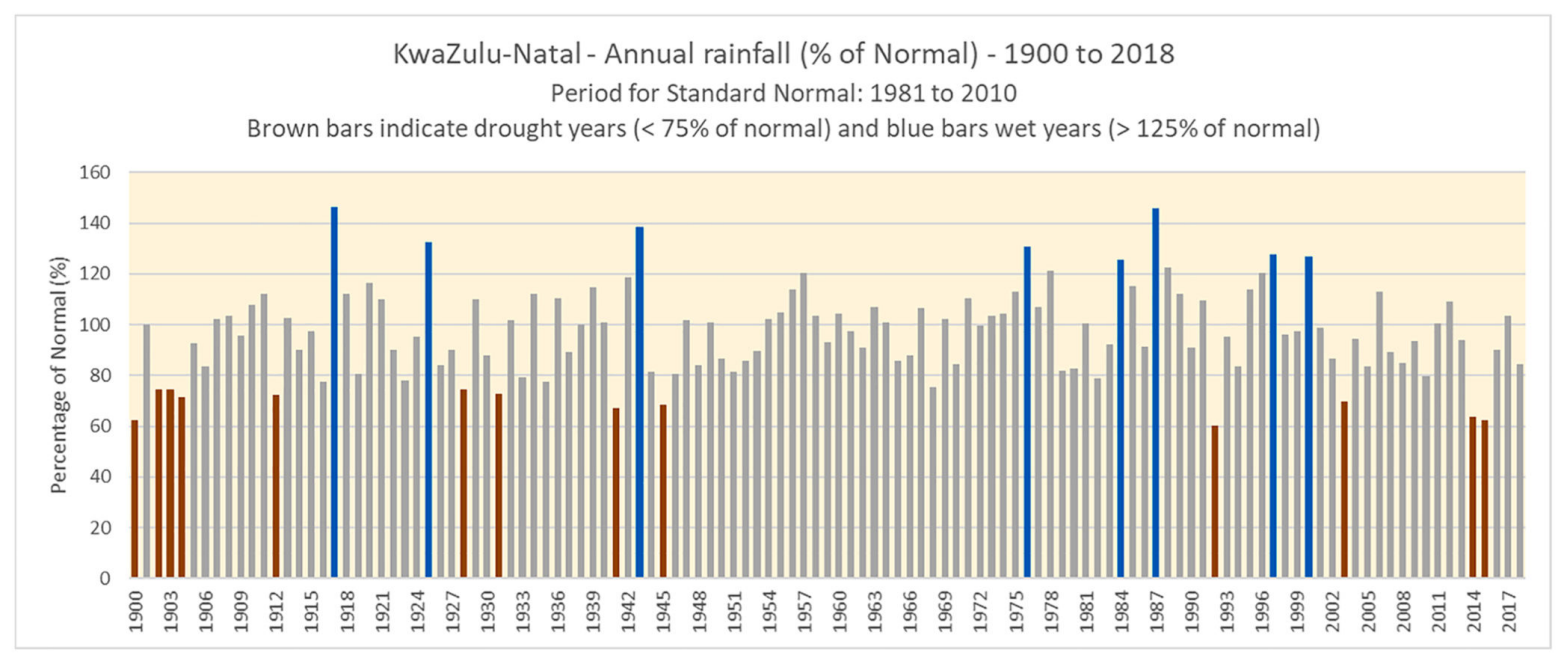
Standard Precipitation Index for KwaZulu-Natal, 1900–2018 (Source: Climate data archive of the South African Weather Service).

As Fig. 1 shows, KZN’s experience of the 2015-16 drought started in 2014 when the province declared a provincial state of disaster (COGTA, 2018; Starkey, 2017). This was due to a reduction in average annual rainfall and water reserves, dried-up rivers and dams, increased cattle and crop death and hunger affecting economic growth, individuals and families who suffered losses and were also vulnerable to other drought-related challenges (Corke and Whittles, 2015; Hlahla and Hill, 2018; Mthembu and Zwane, 2017). Consequently, our study of the interaction between drought and HIV treatment adherence is critical to understanding the combination of factors that could lead to suboptimal HIV treatment adherence.

## 4 Methodology

### 4.1 Qualitative approach

The ‘systems thinking’ adopted for this study is embedded within the broader case study approach and interpretivist paradigm. This enabled us to explore the varying and complex dimensions characteristic of the experiences of PLHIV in uMkhanyakude vis-à-vis the 2015 drought and individuals’ adherence to HIV treatment. Central to this case study approach is the focus and interest on individual cases – PLHIV – and the inherent complexities and non-linear modalities of understanding the participants and their challenges. (Hyett, Kenny, and Dickson-Swift, 2014; Stake, 1995; Anderson et al., 2005). This in-depth focus, aimed at multiple factors entangled in a web of plausible mutual influence, is a major advantage of this approach for our study (Yin, 1999). The interpretivist/constructivist paradigm here reinforces the core direction of this paper in that it allows us to analyse and interpret, drawing on available data, how drought operates through a complex system of interconnected factors with plausible impacts on the adherence to ART of PLHIV.

The research team consisted of an interdisciplinary and multicultural blend of experts in population health, HIV medicine and epidemiology (CI, FT, HM, MS, WH), climate change and the environment (DK, SAK), and the social sciences (JS, NN, OA, KO, SN).

### 4.2 Study sample

Fieldwork and data collection were conducted between August 2019 and June 2020 using a combination of face-to-face in-depth Interviews (IDI) and telephonic in-depth interviews (TIDI) [due to Covid-19 regulations]. The IDIs and TIDIs were conducted with two cohort of PLHIV registered for ART in the local clinics and captured within the Africa Health Research Institute (AHRI) Population Intervention Programme (PIP) database. These comprise those who were still in care hereafter *In-Care (IC) -* and those who had fallen out of care – hereafter *Out-of-Care (OC) -* and captured in the database as lost-to-follow-up.

Participants were randomly selected from PIP databases. Out of the 500 individuals in the first cohort, 16 (IC) participants were interviewed. In the second (OC) cohort, from a sample of 675 individuals on the lost-to-follow-up database (de-identified by the Research and Data Management department at AHRI), 84 individuals were dead, 19 had no PIP household membership. Of the remaining individuals (n = 545) in the database, 27 had no residency status, nine had never been visited by AHRI fieldworkers, five refused to participate in PIP and some others (n = 244) had migrated outside the PIP surveillance area. Consequently, the remaining (n = 287) constituted the sample from which eleven individuals accepted to participate. In total, 27 IDIs were conducted with PLHIV (IC and OC).

### 4.3 Data collection

Interviews were conducted in the local language (IsiZulu) by a social science research assistant (SN) with qualifications in environmental science and trained in qualitative research methods. The interviews were audio-recorded, transcribed and translated by a team of experienced social science research assistants. The interviews were conducted, with participants’ consent, using a topic guide with probing of participants’ experience of, response to and management of their HIV treatment and care during the 2015 drought. Interviews lasted approximately an hour. Reflective notes written daily at the end of interviews about field experiences were used to contextualise and augment participants’ responses. The translation of the interviews was cross-checked and quality controlled by trained research assistants, a data quality coordinator, and a post-doctoral fellow (KO) in the Social Sciences Core department. CI, KO, SAK and DK with the support of the larger team of experts interrogated emerging data to ensure that engagements between SN and study participants addressed the required research questions. The interview records and transcripts were stored on the AHRI server which is password protected. All study staff were trained in Health Research Ethics and confidentiality.

### 4.4 Data analysis

We carried out thematic analysis of the interviews. We used pseudonyms to de-identify individual participants’ transcripts in line with the standard operating procedures within the Social Sciences Core Department at AHRI to ensure anonymity, safety and confidentiality of participants and their views. Translated transcripts were coded using Nvivo Pro 12 (QSR International), and the major themes that emerged were organised in hierarchies drawing on the structured design of the study to identify the economic, social, and demographic impacts of drought. Organising the data along broad thematic areas and identifying sub-themes was vital in displaying some of the intrinsic issues that emerged from the interviews drawing on the commonalities between and within the responses of participants. The systems diagram was used to demonstrate the complex pathways linking drought-related sub-themes and HIV treatment adherence drawing on our findings. The study is reported according to the Standards for Reporting Qualitative Research (O’Brien et al., 2014).

### 4.5 Ethical consideration

This study was approved by the Biomedical Research Ethics Committee of the University of KwaZulu-Natal, South Africa (BREC Ref: BE004/19) and the research governance and ethics committee of the Brighton and Sussex Medical School (Ref: ER/BSMS9B5G/2). Informed consent of participants was obtained prior to their participation and pseudonyms are used in reporting the findings.

## 5 Results and discussions

### 5.1 Characteristics of participants

Twenty-seven PLHIV were interviewed (15 females: median age, 39.5 years (IQR 29.5, 48.8)) most of whom (n = 22) reported having some formal education including high school. 17 of them reported being unemployed and dependent on government social grants – only four reported having full-time employment while others reported losing their “piece” or part-time jobs, with which they supported themselves and their families, during the 2015 drought.

### 5.2 Economic, social and demographic impacts

From the interviews, we identified three interdependent themes describing the economic (disrupted income, livelihoods, and food systems), social (water access, hygiene and sanitation challenges), and demographic (human mobility) impacts of drought on PLHIV in rural KZN. These themes encapsulate their everyday challenges and indicate how they understood the drought to have disrupted their livelihoods-due to its intensity and consequences – with plausible effects on HIV treatment and care.

#### 5.2.1 Economic impacts

##### 5.2.1.1 Disrupted income and livelihoods

Disruption of participants’ livelihoods (sources) due to the 2015 drought was a major cross-cutting finding. The majority of participants’ experiences reaffirmed well noted drought-related disruptions on income, employment and (agricultural) production (Hlahla and Hill, 2018; Mthembu and Zwane, 2017). Participants’ challenges with loss of assets, crop or production failure, selling-off assets due to disrupted incomes, additional financial pressures and other associated spill-over effects were contextualised in an environment characterised by preponderant small-scale agriculture and animal husbandry. A woman highlights the large-scale losses suffered by people regarding cattle, goats, and poultry – some of which were the main sources of individual and family income:

The thing I remember the most about the drought is that cows were dying, especially when walking on the road you see that a cow has collapsed, it has lost energy. When they start saying there is a dead cow there um, you also see the cow’s body – it lost weight. [28 years, IC]

With continued deficit in rainfall and soil water, rivers and dams drying out, the hitherto green fields where livestock grazed withered causing hunger, starvation, and thirst for livestock. With community taps largely unreliable to support families and their livestock, people including cattle owners, were forced to search for milder locations or distant water sources. Elaborating further, another woman noted:

The ones [people] with livestock … find that a cow just sits down and dies. You realise that cows are unable to stand on their own; they lose strength because they [walked with their owners] get water far away from Baswazini… [to Dutch, a location outside Baswazini]… They say that the cattle would return already tired and thirsty. because of the long distance [42 years, IC].

Cattle weariness here emanates from searching for distant water and forage sources, including getting stuck in river mud and possibly falling ill. With many hungry, weak, and possibly infirmed livestock, and faced with mounting financial difficulties, losses became inevitable as well as the economic and psychological implications for individuals, who as Zulus, view these cattle as generational wealth, sources of business exchange and engaging with their ancestors (Shava and Masuku, 2019; MacKinnon, 1999). Losing this crucial component of individual and family identity elicited an intense emotion from a 51-year-old woman who declared: *“I was sad because I knew that livestock is the cornerstone of a home. They say it is not a home [without livestock]“.* By linking the livestock to the home, the participant reaffirmed how disruptive drought-linked livestock losses were for people’s economic sustainability.

Further compounding the widespread drought-enforced assets loss, people experienced spill-over effects such as additional financial difficulties as unskilled workers and artisans found work difficult to come by as (potential) employers could not afford to secure water during this time. A male artisan’s experience demonstrates this point:

Yes, somehow it [drought] has affected my income [as a builder/bricklayer] because if a person [I work for] does not have water, he/she waits for a long period of time while waiting to get water so that I will proceed to build his/her house. If there is no water, I do not work, and sometimes you find that a person I am working for he/she also does not have money to buy water. [45 years, OC]

With both parties, employer and artisan, negatively impacted, people (including PLHIV) who may have already lost livestock were also consigned to precarious labour, unable to earn to support their families and their (potential) medical needs. People who attempted to secure their endangered livestock, were faced with additional financial management decisions – to spend on securing their livestock through buying feed, water and procuring regular inoculation while also providing for family needs or to prioritise one need above others. A male participant explained this conundrum:

… when the grass is gone, and the water is finished, that means the livestock is not eating, it is not drinking, eh, it will die. If you want to sustain them… you have to buy grass. This is the money you are supposed to buy food that will be eaten by you with family members, (but) you are buying grass that the cows eat; yet the livestock ends up dying. [31 years, IC]

Indeed, these stressful circumstances, have been shown (Clarke et al., 2012; Coppock and Desta, 2013), to force people to sell off (other) assets to mitigate drought-impacts. The participant notes that it was *“… better to sell them [livestock] before they die in front of you”* (31 years, IC), but selling off their goats or cows to forestall against total loss also posed a social dilemma for people given their cultural attachment to cattle. They had to make the difficult decision considering the opportunity cost associated with keeping the livestock healthy and alive, bearing in mind another participant’s observation that the drought *“… had a lot of impact because livestock … need its medication and the medication is expensive at the stores…”* (Male, 39 years, OC).

What is exposed here is the deluge of interlinked consequences imposed by drought on PLHIV, their families and society. The risks posed transcended livestock losses to other livelihood impacts such as precarious labour, unemployment and additional financial pressures on them, especially in the area of purchasing grass (animal feed), vaccinations and animal treatment, including water purchase. These additional financial pressures resulting from drought’ s impacts on water availability and vegetation growth, may have forced individuals to weigh up these competing priorities in the face of multiple forms of adversity converging at this time of more extreme shortages. We argue that such a situation potentially disrupts health-seeking behaviour in favour of livelihood sustenance.

##### 5.2.1.2 Disrupted food systems

Food security entails the ability to produce, have access to affordable food, and for the food to be nutritious and safe for consumption (Clover, 2003). Drought can affect this through crop failure, death or livestock losses that impact on individual and household capacity to purchase food when crops fail (Derbile et al., 2016; Gwatirisa and Manderson, 2009; Twongyirwe et al., 2019). A 34-year-old female’s evaluation of drought impact on household food systems emphasises both reduced production and, in some cases, severe losses incurred in crop production both for individual/household consumption or for (mostly petty) commercial purposes as well as reduced availability of healthy meal options:

No, it [food] was disturbed because before we could even grow sweet potatoes and taro root *(amadumbe)* to eat; not now, you really cannot. Now we live on the food we buy from the shops… You keep seeing people sick this much it is because of drought. If we still eat like before, the food we grow, may be there would not be this many sicknesses because the food we grow in the gardens is healthy, not these meats with oil/fats. [34 years, IC]

The impact of drought on soil quality and fertility, and insufficient water for consistent manual irrigation, presented many families with challenges that culminated in withered vegetation, crop failure and insufficient food production. This caused increased family expenditure for staples, water and transportation. These additional financial pressures, and limitations to organic food, for many of the participants, culminated in a perception of health risks linkable to unhealthy foods and snacks from the supermarkets. More still, failing harvests and reduced incomes led to feelings of deep frustration with feeling *“… de-motivated” (Female-24years-IC)* and losing *“… energy”* about farming *(Male-29years-IC),* forcing participants out of farming.

As an agrarian society where subsistence agriculture and animal husbandry are very popular, people in Hlabisa relied mainly on their gardens and small farms to provide for their basic (organic) food needs. Those who engage in varying forms of commercial farming, especially livestock, used them to supplement their overall income. But the loss of livestock and crop failures exacerbated individuals’ and families’ precarious economic and health conditions. This explains such frustrations expressed by people especially when considered in the context of additional vulnerabilities like unemployment and HIV.

#### 5.2.2 Social impacts

##### 5.2.2.1 Drought-related impact on population health: water, sanitation and hygiene

Drought affects population health through various pathways including unstable and unsafe water sources, water-borne, stress-related and chronic diseases (Stanke et al., 2013). Participants’ poor financial standing, and indeed their broader society, deepens the associated drought-related health risks as the issues raised highlight water insecurity, sanitation and hygiene challenges that were heightened during this period.

##### 5.2.2.2 Insufficient access to water

Like food insecurity, most participants expressed how disrupted access to, and inconsistent supply of, water threatened their individual and families’ health, and overall wellbeing. Lack of access to water became a major experience within the community since sources like rivers and dams were depleted, community taps were either broken, non-functional or erratic, government-assisted water tankers were unreliable, and most people lacked boreholes to cushion insufficient supply. A man explained that *“…it was difficult to get water because water pumps were damaged due to the large number of people who were coming from other communities to fetch water daily”* (45 years, OC). Two women explain further:

Like here, they open water for us, every day, from Monday to Friday. Saturday to Sunday . there is no water, but when there is no water for two weeks and more, maybe to 3–4 weeks, that is a month, it is obvious that [something is wrong] When you try to contact someone. they say there is no water: Mfolozi river or Mbukiwini river has dried. [28 years, IC]

… sometimes … we will wake up early around 6o’clock in the morning and walk long miles to Madwaleni where there is a borehole and … you will be able to fetch only one bucket of water. You will come back home maybe around 10o’clock because of the long queues and … you are already tired while the water which you managed to fetch will be exhausted within a single day. It is a very long distance that we walk to fetch water because I think we spend about 40 min while going there. [29 years, OC].

The significance of this experience is in the challenge of daily ‘early-bird’ rising – including the elderly and ailing – and walking or travelling long distances in search of water. Due to insufficient portable water, participants were forced to make additional budgets for water purchase from supermarkets, borehole owners, independent water tankers/distributors and individuals paid for assistance to fetch water from slightly operational dams or rivers. These also imposed difficult challenges on participants, including faithfully keeping their clinic appointments or accessing enough water to take their medication (see more in section 6).

Access to sufficient water during the drought in uMkhanyakude exposes deep social inequalities in the uMkhanyakude district. While water is generally scarce beyond the drought period due to the deep-water table in the district (Dlamini, 2018), the more affluent people residing in peri-urban areas have boreholes. Most people in the outlying villages largely lack the resources to install these boreholes but rely on community taps, infrequent delivery by water trucks or going in search of water in the compounds of families that have boreholes (as in the examples above). The alternative entailed buying water tanks for storage when the water trucks come-by or buying water in jerricans and storing for future (measured) household use.

The strains associated with searching for water (sources) for PLHIV on treatment, added to other vulnerabilities like poverty and comorbidities, have potentially serious implications for optimal treatment adherence. For example, as the excerpts below show, in addition to the challenges of taking medication due to insufficient water, people’s likelihood of missing clinic appointments increased as reported by the participant who missed an appointment because she left home early in search of water:

had to fetch my pills, [but] I could not because I had to get up and fetch water, there was no water. [Female, 27 years-OC] I cannot even take my pill properly because in order for me to drink the pill I need to drink water. … it once happened… But then again, I saw that not drinking my pill because of not having a meal is causing problems for me. The problem is of drinking the pill with only little water; they tell us at the clinic that, when taking the pill, it must be taken with a lot of water. [Female, 49 years-OC]

Although some participants tried to remain consistent with their treatment, insufficient water and poor economic conditions constrained their ability to acquire sufficient food and water. Since they had to purchase these, there was the heightened risk of suboptimal treatment adherence and other health challenges attributable to the poor hygienic circumstances that insufficient water and unsafe water sources exposed them to.

##### 5.2.2.3 Sanitation, hygiene, and health risks

Drought-induced water scarcity, including in rivers and dams, imposed sanitation and hygiene consequences on study participants and their communities. Hygiene risks associated with participants’ drought experience is significant relative to available water sources such as rivers and dams where people and livestock in many instances compete for water. Some participants noted:

Sometimes it happens that there was no water for 4 months.. We will fetch water down there [where] the cows drink and defaecate in the water. We end up drinking dirty water. [Female, 24 years-IC]

If there is drought and we go to the clinic because of perhaps stomach-ache, they would ask us where we keep most of our water. When we tell them, they would advise that we pour some Jik (bleach) in there, but we have to avoid using that water on the same day. [Female, 56 years-OC]

Faced with these challenges, participants resorted to various purification strategies – a popular one, we termed *“Jiking”* – involved using bleach (the Jik brand), cement or alum to “purify” unsafe water from rivers and dams. While participants noted that it was somewhat ‘oral tradition’ propagated in the community and in some of the local clinics, this poses health risks. But despite this potential risk, people were forced (by drought-related circumstances) to do this and potentially expose themselves to various health-risks in their quest to avoid water-borne morbidities (like cholera and dysentery). Indeed, faced with widespread poverty and inconsistent external support, such as government-assigned water trucks, the challenges for PLHIV were daunting as they tried to manage multiple needs, including water security needs while battling to maintain treatment routines.

#### 5.2.3 Demographic impact: Human mobility

Mobility, including temporary or permanent migration, constitutes a popular reaction to and mitigation strategy against drought impacts (Bola et al., 2014; Gray and Mueller, 2012; Nawrotzki et al., 2016; Simatele and Simatele, 2015). Within this study’s context, the dominant mobility-linked impacts of drought centred on relocations and movement of livestock, seeking employment outside the study area and challenges linked to seeking water sources for livestock.

##### 5.2.3.1 Attitudes around mobility and immobility

While migration away from drought-stricken locations is a widespread idea, the finding in this study shows nuanced reactions by those affected by the drought, including PLHIV. Many participants’ stance that they did, and would, not relocate due to the drought despite its negative impacts on their lives and livelihoods is reinforced this woman’s observation that *“people from here love this place, they will never leave”* (42 years, IC). Despite this position, other participants held the opposing view – or confirmed knowing someone, family, or community member, who left the area to escape the drought:

There is no one here around me [who moved] … But I have heard that there are people who have moved in the other places because of drought … [They moved] to Dukuduku but I cannot reveal their names but yes there are people that I know of who moved to Dukuduku for their livestock… to survive [Male, 48 years-IC]

Mobility out of this drought epicentre, as noted by participants (including some who had not intended to migrate) was centred on attempts to seek out forage for livestock or due to the urgency of safeguarding their livestock from drought devastation. Though an economic decision, this move was borne out of the deep-seated importance of livestock as the cornerstone of the Zulu household. Additional reasons linked to migratory activities, such as sending children to relatives and seeking employment opportunities in the urban areas, also entailed people’s calculation of drought’s economic impact on family and household members. The following excerpts are instructive:

… there are those that I know that left here from Somkhele to Dukuduku because they said the area of Dukuduku has rain; drought does not reach there, while they have livestock, so they decided to leave and go build at Dukuduku. [Female,28 years-IC]

Yes, my sister there are, … [people who moved]. No, do you know my sister, they leave when a person is going to hustle, to go and try somewhere where they can try life, where they can get a right life so that they are able to make a living you see … like in the big cities. I have also tried that. in Johannesburg. I wanted to go and try a life there… [Male-39 years-OC]

These perspectives present a nuanced outlook on participants’ attitudes towards migration or relocation from drought-affected communities to other communities or urban areas. What was more interesting pertains to participants who refused to affirm migratory activities or a desire to move away from their drought-affected society despite its intensity and impacts. Such decision(s) to remain instead of out-migrating to escape the drought was contrary to expectation, but psychological and cultural explanations could clarify this phenomenon. For instance, when requested to explain the reasoning behind such decisions (to remain), the issues presented included the fear of leaving home, uncertainties about prospective destinations – including hostility from recipient communities – and their own links to their ancestral homes, symbolised by their family graves:

No, I will stay here sister even if there are droughts where I am it is my home after all, you cannot run away from drought. … It will not help [to move away] because you can relocate and find a huge problem where you have relocated and ending up dead. [Female, 33 years-OC]

No, I will not agree. Leave our family graves as there are just all over the yard? [Female, 49 years-OC]

This rhetoric suggests a level of numbness towards the drought and water insecurity; they seemed to have learned to manage and survive. Consequently, abandoning their homes because of drought made no real sense to them. More than that, it also represents anxiety – the fear of what to expect in the destination should they decide to leave home or what their ancestors would do to them should they abandon their family grave(s). Thus, beyond a feeling of resignation to drought’s existentiality (where escape seemed impossible), they possibly felt locked-in to a dilemma where attempting to ‘escape’ could culminate in losing vital support systems – including their (spiritual) bond with their ancestors.

The ill-treatment of grave sites in Zululand has even been suggested by locals as a reason for the drought – punishment from the ancestors (WoMin, 2017). The ancestors whose graves are marked with tombstones represent the hub of the Zulu society or family, thus abandoning this vital component of their sociocultural life could be dangerous, hence non-negotiable even during severe droughts. Staying put nevertheless exposes them to other water insecurity-risks – hygiene, infectious and non-infectious diseases; food insecurity, income and livelihoods loss and associated and compounded mental health outcomes, all of which affect individual wellbeing. These issues, and the broader health implications of (im)mobility for PLHIV engaged in or disengaged from treatment, are explored further within the interlinkages between drought and treatment adherence in the next section.

### 5.3 Hypothesised relationship between drought themes and ART adherence

**Fig. 2:**
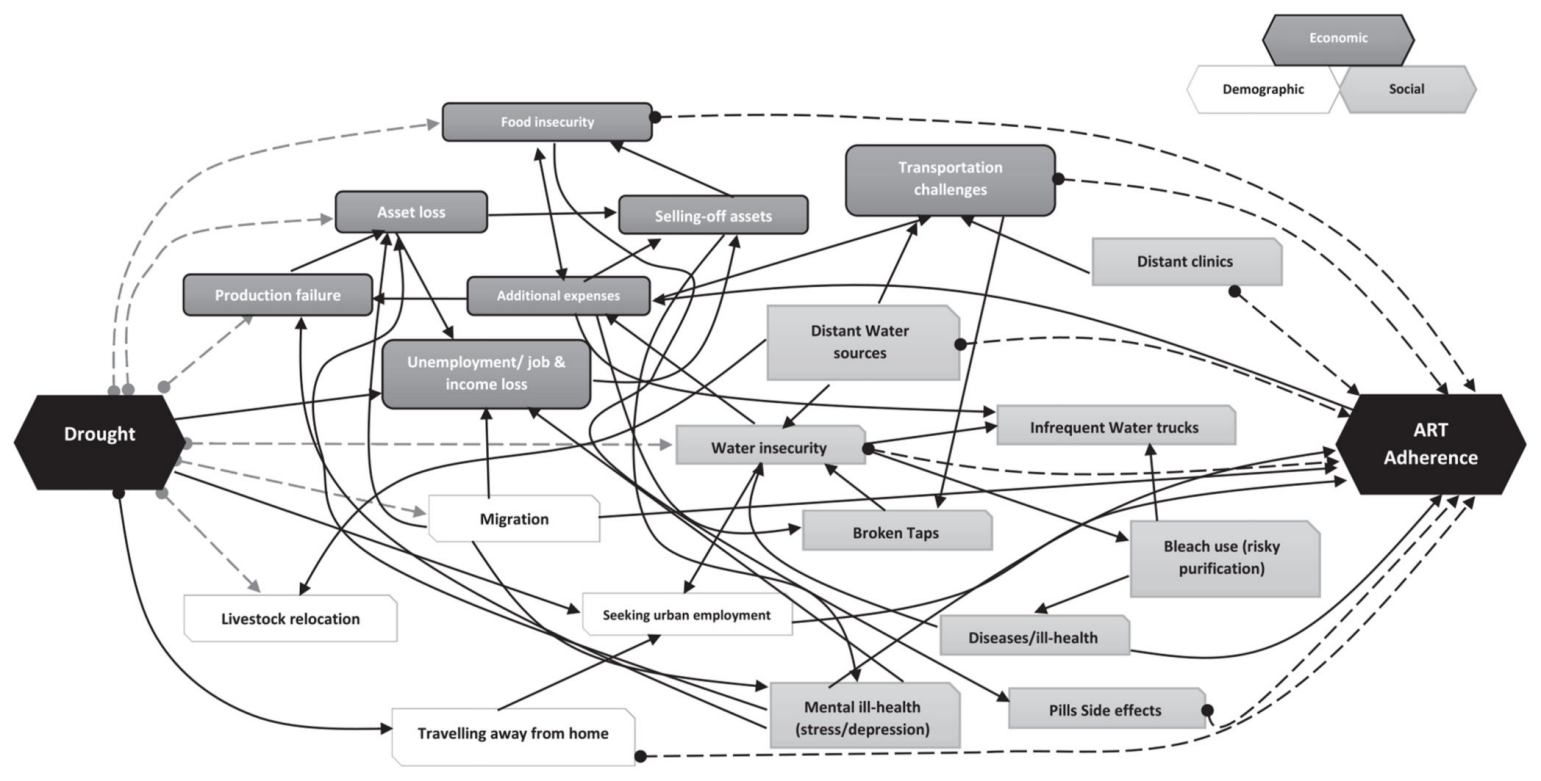
Presents the systems diagram showing the interconnectivities between drought and treatment adherence as conceptualised from interviews conducted with study participants.

Fig. 2 uses a systems diagram to conceptualise the complex nexus of influence and interactions between different factors mediating between drought and suboptimal HIV treatment adherence. The diagram was produced using keywords drawn from interview themes and key findings. These factors or subthemes were connected using arrows through a network of plausible interactions between these variables. The different shades of grey designate the broad socioeconomic and demographic factors ranging from disruptions to livelihoods, income, food systems, population health and human mobility – all of which are well researched factors that contribute towards poor HIV treatment adherence especially in resource poor contexts.

As the interview data suggest, poor ART treatment adherence in Hlabisa is traceable to a complex nexus of interacting socioeconomic and demographic factors which are themselves also potentially exacerbated by the drought duration and intensity. In the diagram, the (double-shaped) grey-dotted arrows moving from drought designate factors (like production failure, assets loss, food insecurity, water insecurity and migration and livestock relocation) that were caused, exacerbated, or simply influenced by the drought – including reactions to the drought. The other black-dotted arrows moving into ART adherence designate some of the major factors that HIV treatment adherence was sensitive to. For example, participants emphasised food insecurity (including the fear of ART side-effects), transportation challenges linked to distant clinics, and more broadly to water insecurity-related issues – all of which are well documented barriers to adherence (Adeniyi et al., 2018; Haberer et al., 2019; Hodgson et al., 2014; Iwuji et al., 2018).

Although some of these factors may already play a role to hinder effective treatment adherence among PLHIV in Hlabisa – the intensity and duration of the 2015 drought may (or would even) have exacerbated the impact of these factors on PLHIV and their ability to manage their treatment effectively. The following excerpts are thus instructive:

Because of the drought I am not able to eat and take my pills. However, when there is no drought I am able to farm and … I take my vegetable, and make whatever I make, eat and drink my pills. [Female, 49 years-OC]

I missed clinic appointment due to shortage of transport fare.… Let me put it like that. It happens that I am saving money to visit the clinic perhaps on the 20th of the month… [only] to find that money is not enough. Kids on the other side are complaining about bread. I then perhaps take this money and buy bread for them and say to myself the date … is still far. When the 20th comes, I have not been able to replace the money I took. As a result, I do not go to the clinic. [Female, 56 years-OC]

Central to these perspectives is how the economic and livelihood conditions of PLHIV – like other members of their society – suffered extreme strain during the 2015 drought. More rapid and intensive production failures, death and or loss of livestock, crops, income, employment and other sources of livelihood culminated in – and in some cases exacerbated – food insecurity, insufficient funds to travel to distant clinics (Adeniyi et al., 2018; Ehlers and Tshisuyi, 2015) and or purchase water, especially when community taps were broken, and water sources were also distant. There is the belief that taking ART on an empty stomach will exacerbate side effects, hence individuals skip their medications when there is no food (Weiser et al., 2012).

Previous studies have demonstrated how drought devastations force people – farmers, herders and households – to sell-off assets to off-set associated additional financial pressures (Lawson and Kasirye, 2013; Quinn et al., 2011). In Hlabisa, PLHIV, many of whom are household breadwinners, had to bear this additional burden of increased cost of food, water, and transportation due to water insecurity and access challenges.

Drought-enforced depletion of soil water/nutrients, rivers and dams with its recorded wide-ranging effects on animal and human health – especially through chronic, infectious and non-infectious diseases, and malnutrition (Stanke et al., 2013) – constrains HIV care (El-Khatib et al., 2011). In Hlabisa, this was heightened by infrequent water tankers and broken taps, forcing PLHIV to go in search of distant and often unsafe water sources like dams and rivers where cattle drink from and people dispose-off sanitary materials. As participants noted above, PLHIV woke too early, walked long distances, and missed clinic appointments due to searching for water. Their reliance, in other instances, on risky water purification methods was also risky for their overall health.

Although some participants delinked drought from poor treatment adherence – like a 51-year-old female who claimed not to have *“… heard that there are (people) who could not reach the clinic because of the drought, because a person goes to the clinic to fight to get their health”* – the complex associated consequences to livelihoods were often dire. In such potential jeopardy, studies show that people usually moved, relocated their livestock or family members as a mitigation strategy (Bosongo et al., 2014; Eyassu et al., 2016; Nawrotzki et al., 2016). This natural instinct to survive or cope with drought through temporary or permanent migration – or simply by travelling out of the drought area (Bosongo et al., 2014; Nawrotzki et al., 2016) – also can have dire consequences on treatment adherence (Iwuji et al., 2018) as the excerpt below suggests:

At some point I was not residing here. I was residing [elsewhere] … where it was discovered that the pill is not effective in my blood system… because I had not been taking my pills… Yes [I stopped taking my pills] … It was a long distance that I could [not] come back here [Mtubatuba] at month-end to refill my treatment… It is because the piece job was over [now], so I was compelled to come back here. [Male, 42 years-OC]

So, despite the reluctance of some people to migrate, owing to their ‘home-attachment’ and the uncertainties surrounding migration, the economic conditions that forced others to move to urban areas like Durban and Johannesburg in search of employment, or in search of forage and water sources for their livestock, can contribute to falling – even briefly – out of care (Adeniyi et al., 2018). Although in our sample population, only a few people (above) confirmed missing treatment due to employment commitments, travelling for work (in Johannesburg or elsewhere) or moving from a different area to the study context or vice versa, it is not exactly clear how well the drought contributed to this. One thing is certain, however: some of those who did move – like a 35-year-old male who “totally stopped” treatment because the regimen’s drowsy effects threatened his life and work as a bodyguard – were PLHIV who engaged in circular migration to support their families financially to mitigate the drought’s additional financial strain. For PLHIV who did not relocate, they continued to risk drought-extended challenges: precarious livelihoods, water and food insecurity and associated mental and physical health constraints that studies have suggested to be detrimental for effective treatment adherence.

## 6 Study limitations

Measuring or timing drought onset is challenging because its impacts accumulate overtime – only fully appreciated by its devastating effects on societies (Stanke et al., 2013). Consequently, we focused more on PLHIV’s experiences within the 2015–16 period when the government declared the drought emergency between 2014/15-16. Though crucial, spatial discussions about what constitutes a drought was not investigated.

Due to the centrality of mobility in drought studies (Low et al., 2019) and ART adherence literature, especially in South Africa (Adeniyi et al., 2018; Iwuji et al., 2018), we expected strong sentiments of drought-enforced mobility with linkage to HIV treatment. However, if drought caused massive out-migration, our sample population would have missed individuals who out-migrated. Also, many participants’ hesitancy towards migration, despite the drought, suggests a cautious argument about linking drought, forced-migration and non-adherence. That notwithstanding, the few cases of movements including by PLHIV helps to contribute to the broader systems thinking that provides a plausible explanation about the connections between the diverse variables and factors, even if at a theoretical level and based upon existing migration-treatment adherence literature.

## 7 Recommendations and conclusion

To our knowledge, this paper is the first to link the physical, psychological, and cultural impacts of drought on PLHIV. The socioeconomic and demographic factors which we explored in terms of how their interaction with drought exacerbated existing, and caused additional, challenges and competing priorities that posed direct and indirect threat to PLHIV’s continuum of care. This was mediated through a range of interlinked physical and mental stresses to their lives that impact on their ability to adhere to ART. These range from restricted food and water intakes during and after drought events interfering with the process of taking medicine through migration reducing access to public health services and the loss of identity through livestock losses. The recurrent nature of drought in uMkhanyakude provides an opportunity to, more effectively, identify local drivers of vulnerabilities (such as ground water table) and information sharing gaps for better planning and intervention.

Pervasive poverty, precarious labour, poorly maintained public water delivery systems, and poor service delivery in general are some of the sources of increased vulnerability in uMkhanyakude. Addressing these issues will prove useful towards reducing the pressures that PLHIV face in managing their health. Effective drought relief interventions like cash support, borehole installations, food banks and consistent water truck presence in the communities would lessen the competing priorities over the disposal of minimal resources between water purchase, transportation, feeding and attending to treatment. Such interventions would benefit from incisive, inclusive, and well-targeted local insight and engagement as regards important areas and issues of priority devoid of elite capture. Consequently, interventions to improve the health and wellbeing of PLHIV should not just focus primarily on HIV but should adopt a whole systems approach to improving public health in general such as ensuring water and food security.

A recent study recommended more male-targeted interventions to enhance suppressive ART with a view to reducing new infections among women in uMkhanyakude (Vandormael et al., 2019). This is crucial especially considering that circular migration, poverty and livelihood challenges, linkable to drought-experiences, are possibly inimical to the optimal level of care required to achieving suppressed viral loads among men.

There is no gainsaying that droughts impact livelihoods as seen through crop failures, production losses, death of cattle and or their reduced productivity as well as a total destruction of individual and community livelihoods. By imposing a myriad of physical and mental health challenges (stress, anxiety, depression) on people, mitigation strategies like mobility – migration – begin to take hold as people seek out avenues to survive. The associated consequences of both direct impacts on livelihoods, health and associated coping mechanism – as the study reveals – can culminate in situations for individuals that are detrimental to treatment adherence.
